# Estudio de la Codificación Exitosa de Memoria Emocional con Resonancia Magnética Funcional: Un Metaanálisis que Compara la Prominencia Afectiva de las Palabras Frente a las Imágenes

**DOI:** 10.31083/RN46063

**Published:** 2025-08-26

**Authors:** Eithan Kotkowski Baca, Sandra Azareli García Velázquez, Peter T. Fox Selby

**Affiliations:** ^1^Research Imaging Institute, University of Texas Health Science Center at San Antonio, San Antonio, TX 78229-3900, USA; ^2^Departamento de Neurología, University of Texas Health Science Center at San Antonio, San Antonio, TX 78229-3900, USA; ^3^South Texas Veterans Health Care System, San Antonio, TX 78229-3900, USA; ^4^Departamento de Radiología, University of Texas Health Science Center at San Antonio, San Antonio, TX 78229-3900, USA; ^5^Departamento de Psiquiatría, University of Texas Health Science Center at San Antonio, San Antonio, TX 78229-3900, USA

**Keywords:** emoción, memoria emocional, palabras afectivas, fotografías afectivas, resonancia magnetica funcional (RMf), RMf de eventos vinculados, estimación de probabilidad de activación (EPA), circunvolución parahipocampal, lóbulo temporal medial, lateralización hemisférica, metaanálisis basado en coordenadas, emotion, emotional memory, affective words, affective pictures, event-related fMRI, funcitonal magnetic resonance imaging (fMRI), activation likelihood estimation (ALE), parahippocampal gyrus, medial temporal lobe, hemispheric lateralization, coordinate-based meta-analysis

## Abstract

**Antecedentes:**

Los estudios de resonancia magnética funcional (RMf) que exploran la codificación de la memoria emocional suelen emplear diseños experimentales basados en eventos con estímulos en forma de palabras o imágenes. Investigaciones previas han sugerido una especialización hemisférica diferencial para estos tipos de estímulos, pero hasta ahora no se ha realizado un metaanálisis que compare directamente los sistemas neuronales implicados en ambos.

**Métodos:**

Se realizó un metaanálisis utilizando estudios de RMf con diseños de eventos vinculados revisados por pares. Se aplicó el método de Estimación de Probabilidad de Activación (EPA) con el software GingerALE para comparar activaciones cerebrales asociadas a la codificación de estímulos emocionales visuales presentados como palabras o fotografías. Se evaluaron tres contrastes: fotografías > neutro + control, palabras > neutro + control, y solapamiento de ambos.

**Resultados:**

Las imágenes evocaron activaciones bilaterales en el parahipocampo medial, mientras que las palabras mostraron activación lateralizada en el parahipocampo izquierdo. El análisis de solapamiento reveló una región común en la amígdala parahipocampal. Los tres contrastes produjeron activaciones significativas en regiones clave del lóbulo temporal medial implicadas en la memoria emocional, incluyendo el hipocampo y la amígdala.

**Conclusiones:**

Ambos tipos de estímulo activaron redes temporales mediales especializadas en la codificación de la memoria emocional. Las palabras activaron específicamente regiones lateralizadas al hemisferio izquierdo, mientras que las imágenes mostraron un patrón bilateral con predominio izquierdo. Este estudio proporciona la primera evidencia metaanalítica de una diferenciación medio-lateral en la circunvolución parahipocampal según el tipo de estímulo emocional.

## Abstract (en)

**Background:**

Functional magnetic resonance imaging (fMRI) studies examining emotional memory encoding often use event-related designs with stimuli in the form of words or pictures. Prior research has suggested differential hemispheric specialization for these stimulus types, yet no meta-analysis has directly compared the neural systems involved in each.

**Methods:**

A meta-analysis was conducted using peer-reviewed, event-related fMRI studies. The Activation Likelihood Estimation (ALE) method was applied via GingerALE software to compare brain activations associated with the encoding of affective visual stimuli presented as either words or photographs. Three contrasts were assessed: pictures > neutral + control, words > neutral + control, and overlap between both.

**Results:**

Picture stimuli elicited bilateral activation in the medial parahippocampus, while word stimuli produced left-lateralized activation in the lateral parahippocampus. The overlap analysis identified a shared region in the parahippocampal amygdala. All three contrasts revealed significant activations in key medial temporal lobe (MTL) regions involved in emotional memory, including the hippocampus and amygdala.

**Conclusions:**

Both stimulus types engaged medial temporal networks specialized in emotional memory encoding. Word stimuli selectively activated regions lateralized to the left hemisphere, whereas picture stimuli produced bilateral activation with a leftward bias. This study provides the first meta-analytic evidence of a medial-lateral differentiation in the parahippocampal gyrus based on emotional stimulus type.

## 1. Introducción

La accesibilidad para estudiar impedimentos neurocognitivos sin tener que acumular casos clínicos de lesiones al cerebro o extirpar porciones del cerebro ha avanzado dramáticamente desde la aplicación de resonancia magnética para producir imágenes del cerebro. En particular, los estudios por resonancia magnética funcional (RMf) sirven para destacar y visualizar la actividad dentro del cerebro temporalmente según el estímulo proporcionado al participante, sea una actividad, estímulo motriz, sensorial, o cognitivo. Este método, conocido como “dependencia del nivel de oxígeno sanguíneo” (“blood oxygen level dependency” en inglés) utiliza los principios de oxigenación de células en el cerebro por medio de la transformación de la molécula de la hemoglobina.

Ciertos experimentos de eventos vinculados utilizan la RMf para investigar diferencias explicitas entre la acción de codificación vinculada con el tiempo al comienzo de un estímulo. Con esto se puede investigar la actividad cerebral retrospectivamente para visualizar al cerebro en el momento en que un estímulo fue presentado, y con eso diferenciar los estímulos recordados y olvidados. Por ejemplo, si a un participante se le presenta una lista de cien palabras al estar dentro de la RMf, y nada más recuerda veinte, se puede retroactivamente medir el nivel de actividad del cerebro en ciertas regiones anatómicas y analizar las diferencias entre los estímulos que fueron recordados y aquellos que fueron olvidados. Con respecto al presente estudio, la RMf permite que los experimentos de eventos vinculados identifiquen actividades correspondientes a codificaciones exitosas y estímulos recordados con actividades implementado la memoria emocional. Este tipo de diseño ha sido utilizado para estudiar cómo diferentes regiones anatómicas del cerebro se especializan. Además, otros estudios parecidos han demostrado continuamente que la amígdala se activa cuando se realizan codificaciones exitosas de la memoria emocional en comparación con codificaciones de la memoria neutra [1]. Otros estudios neuroimagenológicos utilizando modelos de conectividad funcional han respaldado estas observaciones con métodos estadísticos usando estudios que analizan el nivel de coactividad entre el hipocampo y la amígdala [2, 3, 4, 5, 6, 7].

En este estudio, asumimos la especificidad del contenido del hipocampo y el lóbulo parahipocampal izquierdo según estudios que abordan la especificidad del mundo semántico dentro de estas estructuras cerebrales [8, 9, 10, 11, 12]. A pesar de que ciertos estudios han concluido esta especificidad, actualmente existe una carencia de estudios que explícitamente demuestren la existencia de la dicotomía izquierda-derecha dentro de la región del lóbulo temporal medio (LTM) y el hipocampo con respecto a la codificación de la memoria emocional visual lingüística en comparación con la fotográfica. Sin embargo, en previos estudios, se ha encontrado una asimetría de las estructuras del LTM con activación lateralizada a la derecha con estímulos visuales no verbales [13, 14]. Asimismo, otros estudios han aludido a lateralizaciones del lado izquierdo en estructuras del LTM asociadas con la memoria semántica [15, 16, 17, 18]. Es por esto que un metaanálisis con estudios de RMf de eventos vinculados es el sistema más apropiado para acabar con dudas.

Decidimos realizar un metaanálisis distinto usando como ejemplo un estudio basado en coordenadas cuya meta originalmente fue investigar la especificidad de regiones cerebrales contrastando los estudios de estímulos afectivos versus no afectivos [19]. Cada estudio y experimento en este metaanálisis (Tabla 1, Ref. [2, 7, 20, 21, 22, 23, 24, 25, 26, 27, 28, 29, 30, 31, 32]) utilizó RMf con un diseño de eventos vinculados reportando la actividad cerebral utilizando estímulos afectivos (emocionales) en forma de fotografías o palabras escritas. La mayoría de los estudios incluidos compararon memoria emocional con memoria neutra utilizando uno de los estímulos, pero en nuestro estudio comparamos los dos diferentes estímulos enfocados únicamente en los estímulos afectivos. Tres contrastes estadísticos fueron calculados: las activaciones según estímulos fotográficos, las activaciones según estímulos lingüísticos, y la combinación de estímulos de fotografías y palabras. Nuestro propósito fue destacar diferencias dentro de y entre las regiones cerebrales asociadas con la codificación de la memoria emocional. Para lograr esto, primero tuvimos que demostrar meta-analíticamente con experimentos de eventos vinculados que existen activaciones al comparar estímulos emotivos fotográficos y lingüísticos independientemente con “neutrales + controles”. Ya habiendo establecido que existen activaciones en el cerebro para los dos diferentes estímulos, pudimos después comparar los estímulos fotográficos con los lingüísticos.

**Tabla 1.** **Experimentos involucrando estímulos visuales en formas de escenografía, objetos, y caras humanas incluidas en el metaanálisis al igual que experimentos involucrando estímulos lingüísticos visuales en forma de palabras escritas**.

1er Autor	Número de Referencia	N (N mujeres)	Experimento: Valencia y Contraste Estandarizada	Estímulo	Plazo de Retención	Tarea de Retención	AME: Aciertos/RC	CE o LTM
Cahill	[2]	23 (11)	Excitación Positiva & Negativa > Neutral	Escenas	2 semanas	Reconocer/Recordar, Saber	Sí/–	CE
Canli	[20]	24 (12)	Excitación Positiva & Negativa > Neutral	Escenas	3 semanas	Reconocer/Recordar, Familiar	Sí/–	LTM
Kensinger	[21]	34 (23)	Excitación Positiva & Negativa > Control	Objetos	30 min	Reconocer	Sí/–	CE
Kensinger	[21]	34 (23)	Excitación Positiva > Control	Objetos	30 min	Reconocer	Sí/–	CE
Kensinger	[21]	34 (23)	Excitación Negativa > Control	Objetos	30 min	Reconocer	Sí/–	CE
Kensinger	[21]	17 (12)	Excitación Positiva > Control	Objetos	30 min	Reconocer	Sí/–	CE
Kensinger	[21]	20 (10)	Excitación Negativa > Control	Objetos	30 min	Reconocer	Sí/–	CE
Mackiewicz	[22]	40 (18)	Excitación Negativa > Neutral	Escenas	2 semanas	Reconocer	Sí/No	LTM
Mickley Steinmetz	[23]	20 (10)	Excitación Positiva & Negativa > Neutral	Escenas	30 min	Reconocer/Recordar, Saber	Sí/Sí	CE
Mickley Steinmetz	[23]	20 (10)	Excitación Positiva & Negativa > Neutral*	Escenas	30 min	Reconocer/Recordar, Saber	Sí/Sí	CE
Rasch	[24]	57 (41)	Excitación Negativa > Neutral	Escenas	10 min	Recordar	Sí/–	CE
Rasch	[24]	57 (41)	Excitación Positiva > Neutral	Escenas	10 min	Recordar	Sí/–	CE
Rasch	[24]	57 (41)	Excitación Negativa > Neutral*	Escenas	10 min	Recordar	Sí/–	CE
Rasch	[24]	57 (41)	Excitación Positiva > Neutral*	Escenas	10 min	Recordar	Sí/–	CE
Ritchey	[7]	13 (7)	Excitación Positiva & Negativa > Neutral	Escenas	1 semana/	Reconocer/Certeza	–/Sí	CE
					20 min			
Ritchey	[7]	13 (7)	Excitación Positiva & Negativa > Control	Escenas	1 semana/	Reconocer/Certeza	–/Sí	CE
					20 mins			
Ritchey	[7]	13 (7)	Excitación Positiva & Negativa > Neutral	Escenas	1 semana/	Reconocer/Certeza	–/Sí	CE
					20 mins			
Ritchey	[7]	13 (7)	Excitación Positiva & Negativa > Neutral*	Escenas	1 semana/	Reconocer/Certeza	–/Sí	CE
					20 mins			
Sergerie	[25]	18 (9)	Excitación Negativa > Control	Caras	5 min	Reconocer	–/Sí	CE
Sergerie	[25]	18 (9)	Excitación Negativa > Neutral	Caras	5 min	Reconocer	–/Sí	CE
Sergerie	[25]	18 (9)	Excitación Negativa > Control*	Caras	5 min	Reconocer	–/Sí	CE
Sergerie	[25]	18 (9)	Excitación Negativa > Neutral *	Caras	5 min	Reconocer	–/Sí	CE
St Jacques	[26]	15 (15)	Excitación Negativa > Neutral	Escenas	45 min	Reconocer con Señas	Sí/–	CE
Talmi	[27]	11 (5)	Excitación Positiva & Negativa > Neutral	Escenas	25 min	Reconocer/Certeza	–/Sí	CE
Talmi	[27]	11 (5)	Excitación Positiva & Negativa > Neutral*	Escenas	25 min	Reconocer/Certeza	–/Sí	CE
Dougal	[28]	14 (9)	Excitación Positiva & Negativa > Control	Palabras	1 día	Reconocer/Origen	Sí/No	CE
Dougal	[28]	14 (9)	Excitación Positiva & Negativa > Neutral	Palabras	1 día	Reconocer/Origen	Sí/No	CE
Kensinger	[29]	16	Excitación Positiva & Negativa > Control	Palabras	1–2 días	Reconocer/Origen	Sí/–	CE
Kensinger	[30]	28	Excitación Positiva & Negativa > Control*	Palabras	10 min	Reconocer/Recordar, Saber	Sí/No	CE
Sommer	[31]	17	Excitación Positiva & Negativa > Neutral	Palabras	5 min	Reconocer/Certeza	No/No	CE
Sommer	[31]	17	Excitación Positiva & Negativa > Control	Palabras	5 min	Reconocer/Certeza	No/No	CE
Kensinger & Schacter	[32]	21	Excitación Positiva & Negativa > Control	Palabras	30 min	Reconocer/Origen	Sí/–	LTM

* Puntajes de reconocimientos corregidos (aciertos menos falsas alarmas); AME, ampliación en memoria emocional; RC, data con reconocimientos corregidos; LTM, lóbulo temporal medio; CE, cerebro entero.

## 2. Métodos

Los métodos del metaanálisis en esta investigación utilizan el principio estadístico conocido como “Estimación de Probabilidad de Actividad (EPA)” — en inglés “Activation-Likelihood Estimation (ALE)”. Este principio utiliza el formato de coordenadas aplicado a varios experimentos de eventos vinculados reportando la actividad funcional cerebral tabulada en los resultados de los artículos publicados en revistas científicas revisados por pares (arbitraje). Los métodos que delinearon cómo los artículos incluidos en este estudio fueron escogidos, filtrados por medio de calidad y análisis estadísticos, se pueden apreciar posteriormente.

Este estudio se basó en la metodología y los criterios de selección originalmente establecidos por Murty *et al*. (2009) [6] en su metaanálisis sobre la codificación de la memoria emocional. Específicamente, adaptamos sus criterios de inclusión basados en coordenadas, estrategias de búsqueda y umbrales estadísticos de ALE utilizando la versión actualizada del software GingerALE (versión 2.3.2, University of Texas Health Science Center at San Antonio’s Research Imaging Institute, San Antonio, TX, USA) y el espacio estandarizado MNI-152.

### 2.1 Búsqueda por Internet y Filtros de Inclusión

Adoptamos criterios estandarizados para recuperar artículos con criterios de inclusión para nuestro metaanálisis (Tabla 1). Usando el artículo de Murty *et al*., 2011 [19] como modelo, usamos los mismos criterios para replicar los criterios de inclusión de los cuales se generaron veinte artículos. De estos veinte, once reportaron activaciones con estudios de RMf utilizando estímulos fotográficos (25 experimentos en total con 292 participantes) y cuatro reportaron activaciones utilizando estímulos lingüísticos (siete experimentos en total con 96 participantes). La mayoría de estos estudios reportaron los focos de actividad usando coordenadas en el espacio del Instituto Neuroimagenológico de Montreal – Montreal Neuroimaging Insitiute (MNI). Debido a esto, todos los focos fueron estandarizados al espacio conocido como MNI-152 para efectuar y reportar los análisis estadísticos subsiguientes.

### 2.2 Métodos para Analizar la Estimación de Probabilidad de Actividad

Dos análisis de EPA principales fueron efectuados: (1) Un análisis para replicar los estudios de Murty *et al*. (2011) [19] utilizando sus 18 estudios originales y empleando los mismos parámetros delineados en su publicación cuyo enfoque fue contrastar los estímulos afectivos con los estímulos neutros y combinando los estímulos en forma de fotografías y palabras. (2) Un análisis subsiguiente contrastando únicamente los estudios empleando estímulos afectivos en forma de fotografías contra palabras.

### 2.3 El Análisis de Estimación de Probabilidad de Actividad

La conformidad espacial entre los focos de actividad agrupados fue calculada con el algoritmo modificado de EPA según Eickhoff *et al*., 2012 [33]. Como se describe en artículos metodológicos previos [34, 35], para poder indicar la incertidumbre espacial reportada en focos de activación dentro de estudios de RMf, la EPA trata a cada foco como una distribución probabilística gaussiana. Las distribuciones gaussianas después son agrupadas al nivel del primer vóxel dentro de grupos seleccionados, y luego contrastados entre otros grupos para crear un mapa EPA dentro de un espacio cerebral común.

El algoritmo de EPA incluye los siguientes avances: limitaciones estadísticas conforme a los efectos de focos individuales a través de experimentos y distintos grupos [35]; un enfoque analítico y basado en datos para crear una distribución nula utilizada en inferencias estadísticas (previamente, un proceso de permutación empírica) [34]; y un enfoque estadístico Monte-Carlo que permite inferencias más precisas al nivel de agrupaciones. Asimismo, según nuestro análisis, el algoritmo de Eickhoff *et al*., (2012) [33] representa lo más actual en métodos estadísticos para investigar metaanálisis basados en coordenadas. Todos los análisis de EPA fueron realizados utilizando GingerALE 2.3.2 (https://brainmap.org/ale/index.html) y el umbral aglomerativo de agrupaciones (Pb 0,01) con una taza de descubrimientos falsos de (pN, Pb 0,01).

### 2.4 El Análisis de Contrastes

Las imágenes de EPA correspondientes a grupos de “fotografías” y “palabras” fueron efectuadas utilizando los métodos mencionados anteriormente y fueron comparadas estadísticamente con varias regiones conteniendo semejanzas y divergencias entre los dos grupos. Esta función se puede encontrar dentro del programa GingerALE. Para corregir las diferencias en los tamaños de los estudios, datos simulados fueron generados al azar utilizando las bases de datos originales y dividiéndolas en particiones del mismo tamaño como datos de entrada [34]. Este proceso fue repetido con 10.000 iteraciones, cediendo una distribución nula con la diferencia entre las puntuaciones de EPA entre los grupos de “fotografías” y “palabras”. Las diferencias observadas en las puntuaciones de EPA fueron después probadas en contra de la hipótesis nula al nivel de cada vóxel, finalmente generando un valor p estadístico al nivel del vóxel representado como vestimento visual con un umbral designado por la taza de descubrimientos falsos y corrección Bonferroni de pN <0,01 y una agrupación de voxeles de 100 mm^3^ mínimos. A continuación, los resultados se superpusieron a la plantilla cerebral “MNI-152” ponderada en T1 en 3D en el espacio de coordenadas de Talairach.

## 3. Resultados

Tres contrastes fueron generados delineando las regiones de activación en el cerebro conforme a la codificación de estímulos visuales en forma fotográfica (fotografías > neutral + control), estímulos visuales lingüísticos en forma de palabras (palabras > neutral + control), y solapamiento de fotografías + palabras. El término “neutral” en este contexto corresponde a imágenes o palabras sin cargo emocional (por ejemplo, la imagen de una cuchara, o la palabra “cuchara”). Asimismo, el término “control” corresponde a una cruz blanca con un campo negro que se entrelaza entre las exposiciones de imágenes o palabras dentro del experimento de eventos vinculados. El propósito de estos contrastes fue diferenciar entre regiones específicas del cerebro asociadas con la codificación de estímulos emocionales, nivel de activación y lateralización. Cada contraste resultó en activaciones estadísticamente significativas en regiones del cerebro implicadas en la memoria emocional. Estas regiones incluyeron el giro temporal inferior, hipocampo, parahipocampo, y otros. Todas las regiones destacadas alcanzaron el umbral de inclusión sobrepasando el valor *p* estadístico de 0,01 (Tabla 2).

**Tabla 2.** **Agrupamientos consistentemente activados a través de los estudios globales (*p* < 0,01, corregido por tazas de descubrimiento falsos)**.

Agrupaciones de Estímulos Visuales: Emocional > Neutral/Control						
Región	Área Brodmann	Hemisferio	Volumen (mm^3^)	*x*	*y*	*z*
Uncus, Amígdala	–	Derecho	2138	20	0	–20
Giro Temporal Inferior	37	Derecho	187	46	–64	0
Giro Supramarginal	40	Derecho	57	48	–38	34
Parahipocampo, Amígdala	–	Izquierdo	3189	–22	–6	–18
Giro Occipital Medio	19	Izquierdo	434	–52	–70	6
Parahipocampo	28	Izquierdo	155	–24	–20	–12
Giro Frontal Medio	10	Izquierdo	45	–2	62	16
Agrupaciones de Estímulos Lingüísticos: Emocional > Neutral/Control						
Región	Área Brodmann	Hemisferio	Volumen (mm^3^)	*x*	*y*	*z*
Parahipocampo, Hipocampo	–	Izquierdo	1970	–30	–12	–14

### 3.1 Contraste 1

**Fig. 1.**
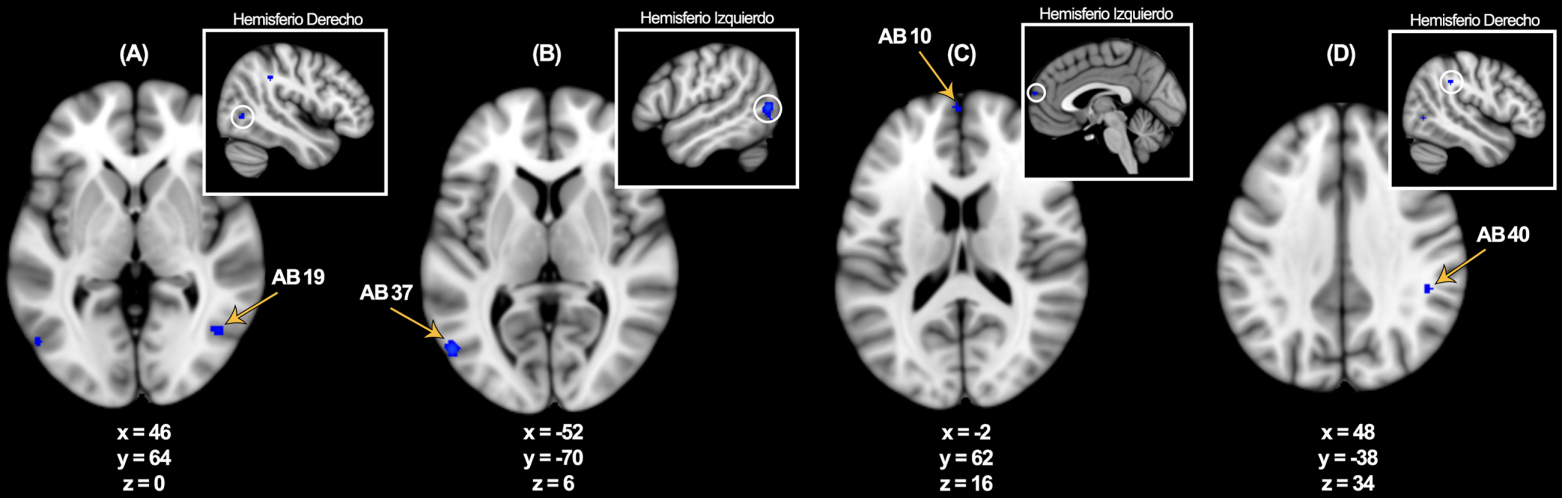
**Regiones demostrando activaciones corticales confiables dentro de la corteza visual (A) y (B), corteza anterior prefrontal izquierda (C), y giro supramarginal en la corteza parietal derecha (D)**. Mapa de activación probabilística para estímulos visuales y lingüísticos utilizando un análisis global (*p* < 0,01, corregido por tazas de descubrimiento falsos) superpuesto en el temple de MNI152. Las coordenadas son reportadas en el espacio Talairach. AB, Área Brodmann.

Fotografías > Neutral + Control: El primer contraste resultó en siete áreas con agrupaciones volumétricas extendiéndose de 45 mm^3^ a 3189 mm^3^. Los volúmenes de agrupaciones más notables fueron las amígdalas, el giro parahipocampal izquierdo, el giro inferior temporal derecho, el giro supramarginal derecho, el giro occipital medio izquierdo, el parahipocampo izquierdo, y el giro frontal medial (Fig. 1). El volumen total de la agrupación dentro del hemisferio izquierdo midió 1441 mm^3^ más que el derecho.

### 3.2 Contraste 2

Palabras > Neutral + Control: El segundo contraste resulto únicamente en una región estadísticamente significativa, pero con activación robusta, localizada en el giro parahipocampal izquierdo con una agrupación que midió 1970 mm^3^ volumétricamente.

### 3.3 Contraste 3

**Fig. 2.**
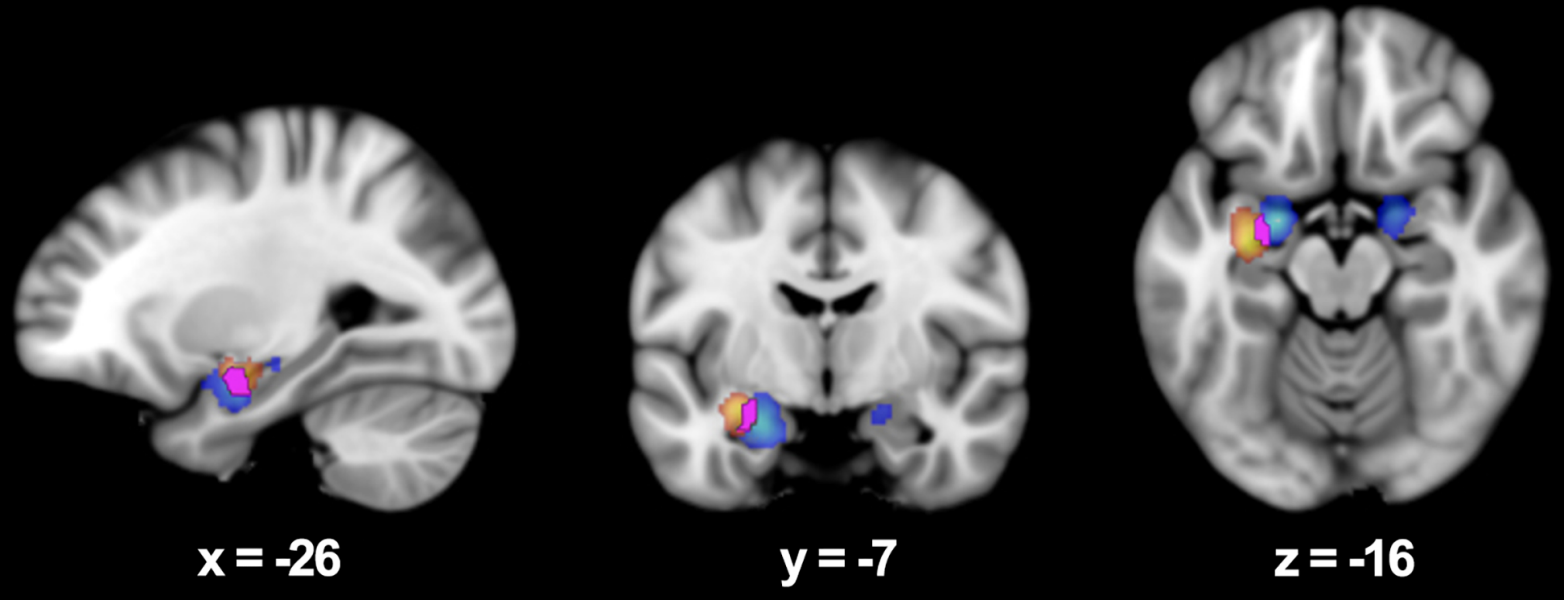
**Área demostrando solapamiento dentro del parahipocampo correspondiendo a estímulos visuales y lingüísticos**. Mapa de activación probabilística para estímulos visuales y lingüísticos utilizando un análisis global (*p* < 0,01, corregido por tazas de descubrimiento falsos) superpuesto en el temple de MNI152. Las coordenadas son reportadas en el espacio Talairach.

Solapamiento de Fotografías y Palabras: Un tercer contraste fue creado para aislar a las regiones sobre posicionadas entre agrupaciones de estímulos lingüísticos y fotográficos. Una agrupación relativamente pequeña de 357 mm^3^ localizada en la región amigdalar parahipocampal fue descubierta (Fig. 2). Los archivos 3D sin procesar corresponden a la Fig. 2, ver **material complementario**.

## 4. Análisis

Las investigaciones meta analíticas de estudios de eventos emotivos vinculados por resonancia magnética funcional (RMf) han demostrado activaciones en redes de regiones cerebrales entrelazadas que contrastan estímulos lingüísticos con no-lingüísticos visuales durante de la codificación de la memoria emocional. Los resultados de esta investigación re-demostraron que evidentemente existe una especialización del parahipocampo izquierdo para estímulos emotivos visuales-lingüísticos (palabras emotivas proyectadas ante una pantalla) y bilateralmente en los dos parahipocampos para estímulos emotivos visuales-fotográficos (fotografías con imágenes representando escenas y objetos escogidas para generar reacciones emotivas).

**Fig. 3.**
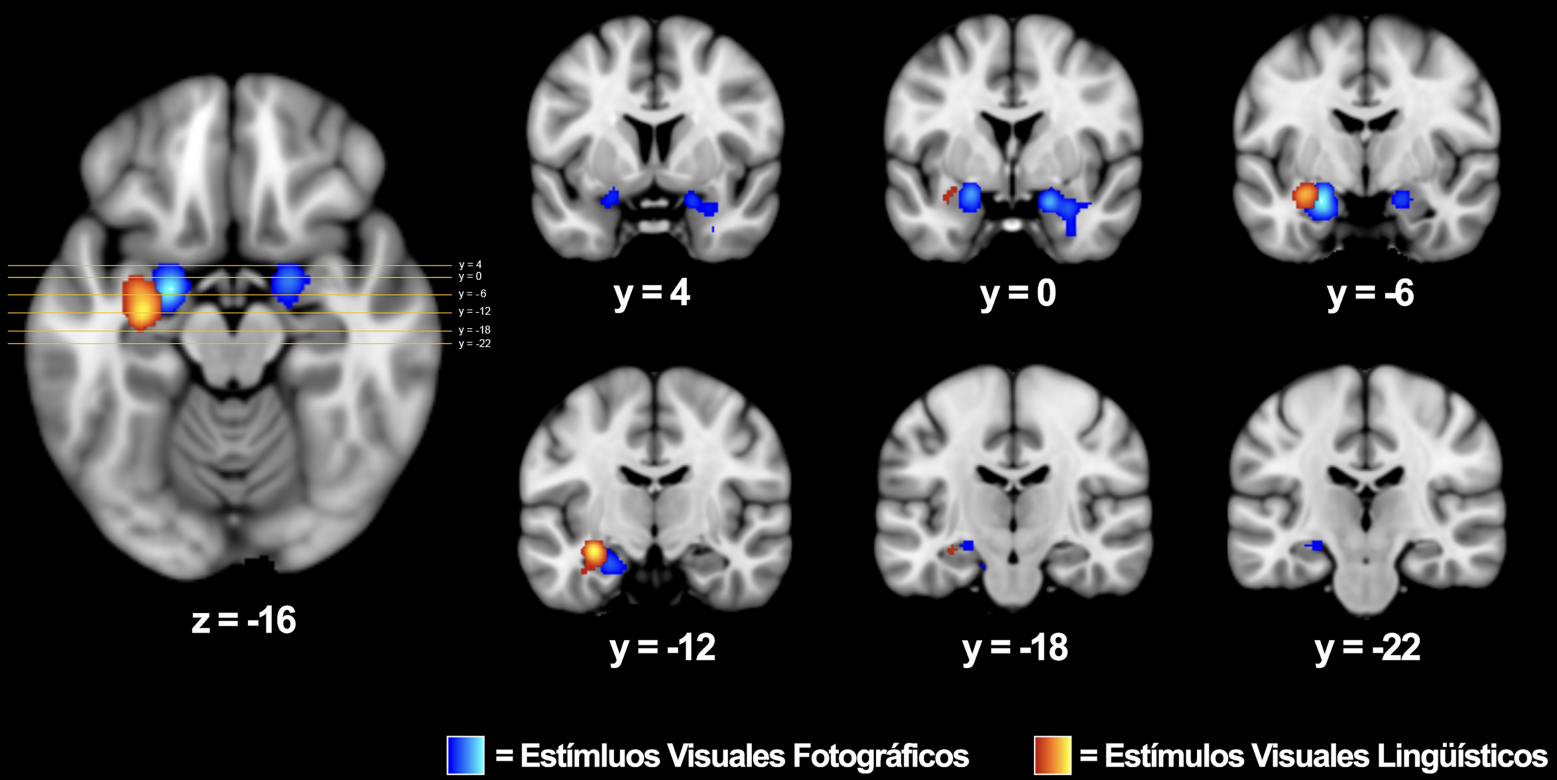
**Regiones demostrando activaciones confiables dentro del sistema de memoria incluyendo a las amígdalas, los hipocampos, y los parahipocampos**. Mapa de activación probabilística para estímulos visuales y lingüísticos utilizando un análisis global (*p* < 0,01, corregido por tazas de descubrimiento falsos) superpuesto en el temple de MNI152. Las coordenadas son reportadas en el espacio Talairach.

**Fig. 4.**
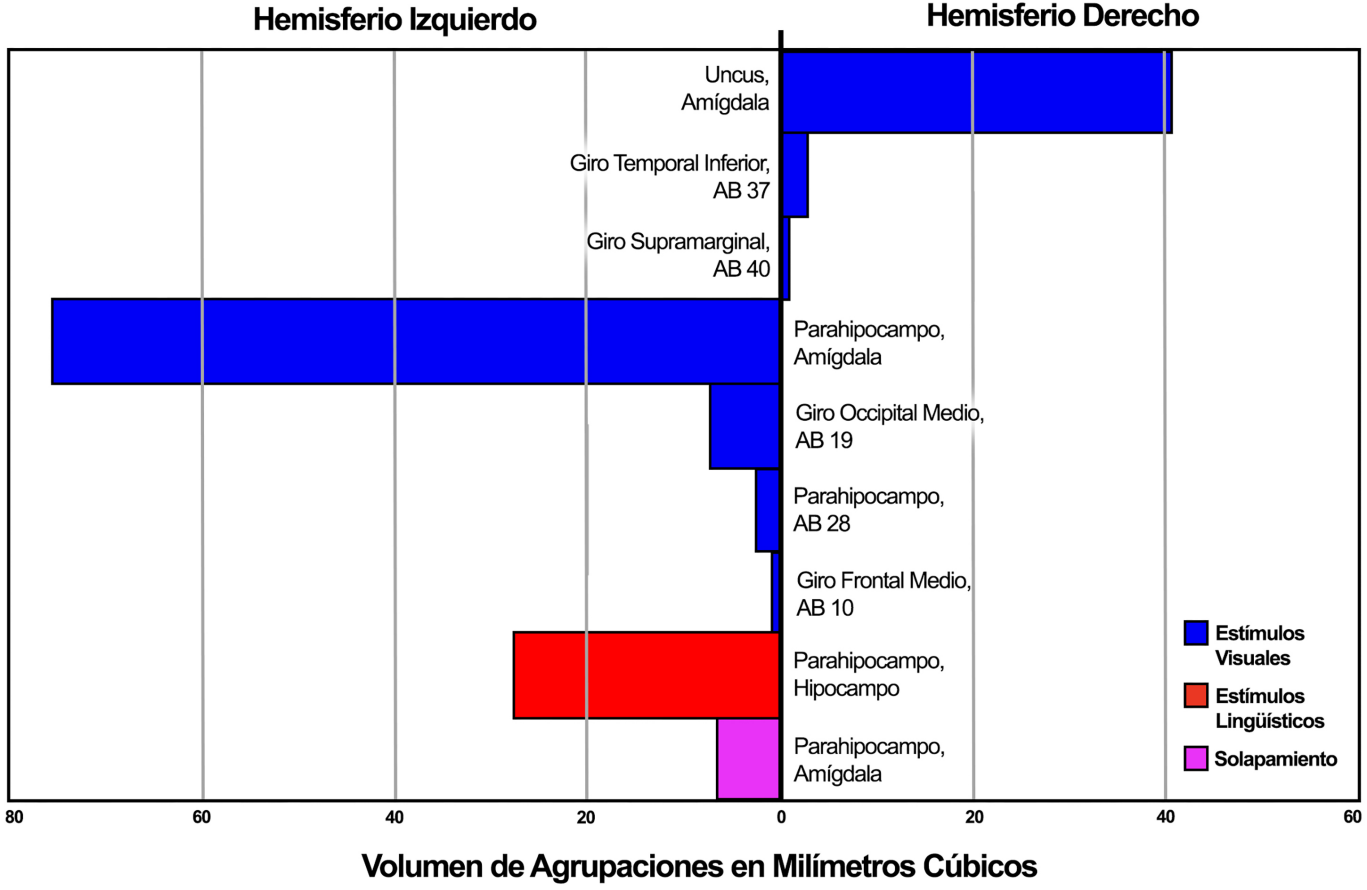
**Grafico demostrando la lateralización de agrupaciones de actividad conforme a su volumen en milímetros cúbicos dentro de las regiones correspondientes**.

Encontramos que los dos tipos de estímulos brindaron regiones de activaciones convergentes robustas en las regiones temporales medias; regiones principalmente responsables de la codificación de la memoria y que incluyen las amígdalas y los parahipocampos. Se encontró una diferencia robusta entre los dos estímulos, en la que los focos de estímulos verbales parecían estar exclusivamente lateralizados hacia el hemisferio izquierdo y su foco estaba más lateralizado que los focos visuales dentro del parahipocampo izquierdo (Fig. 3). Los archivos 3D sin procesar corresponden a la Fig. 3, ver **material complementario**. Las agrupaciones generadas por imágenes fotográficas demostraron activaciones bilateralmente dentro de las regiones temporales medias que incluyen las dos amígdalas y parahipocampos con la agrupación más densa encontrada en el hemisferio izquierdo (Fig. 4).

Debido a su proximidad con la amígdala; el hipocampo, la circunvolución parahipocampal y otras estructuras circundantes han sido implicadas en la formación de la memoria declarativa, lo que hace que la corteza del LTM sea el objetivo de la mejora de la memoria emocional mediada principalmente por la amígdala [36]. Murty *et al*., (2009) [6] descubrió que muchas de las regiones del LTM pueden ser distinguidas por su especialización en el procesamiento y codificación de la memoria emocional. Sin embargo, cuando se trata de identificar regiones cerebrales específicas y la naturaleza lateralizada para los estímulos semánticos frente a los visuales, la hipótesis anterior de que una mayor parte del hipocampo derecho y la región parahipocampal es necesaria para los estímulos no verbales [14, 37] y la izquierda es necesaria para los estímulos verbales se confirmó [38].

Con respecto al proceso de codificación de los estímulos emotivos visuales fotográficas, el descubrimiento más interesante fue que las regiones activadas, aunque estén presentes bilateralmente, ocupan mayor volumen en el LTM izquierdo que en el derecho. Una hipótesis propuesta por Kondo *et al*. (2005) [39] sugiere que esto puede ser debido a que el proceso elaborativo lingüístico se recluta como una técnica mnemónica para recordar estímulos visuales. Es decir, se puede asumir que un componente de la memoria visual es lingüístico y posiblemente principalmente lingüístico ya que el dialogo interno de los pensamientos humanos incorporan estímulos de los sentidos como memoria en forma narrativa.

Aún hay preguntas por responder en términos de cómo el cerebro prioriza estímulos de memoria codificada y cómo la variedad del tipo de estímulos influye en la memoria declarativa. La memoria afectiva (o emocional) puede beneficiar a los investigadores representando un resultado indirecto de la modulación de otros sistemas neurológicos dedicados a la codificación de memoria general, p.ej. atención, memoria de trabajo, y elaboración semántica [1, 5, 30, 40]. Como consecuencia, uno se puede hacer las preguntas ¿cuáles son las circunstancias en las cuales la codificación de memoria neutra y emotiva se desvían? y ¿en qué punto se entrelazan los estímulos visuales y lingüísticos con estrategias mnemónicas como la elaboración semántica?

## 5. Conclusiones

En resumen, encontramos que tanto los tipos de estímulo de imagen como los de palabras produjeron regiones robustas de convergencia de activación en las regiones cerebrales temporales mediales y específicas de la memoria. Las respuestas basadas en imágenes fueron bilaterales (izquierda > derecha) y situadas en la porción medial del parahipocampo. Las respuestas basadas en palabras estaban fuertemente lateralizadas a la izquierda y situadas en la cara lateral del parahipocampo. Se confirmó la especialización establecida en el hemisferio izquierdo de las estructuras temporales mediales para los estímulos verbales. Hasta donde sabemos, esta es la primera demostración de una diferenciación medial-lateral de la circunvolución parahipocampal para estímulos de imágenes y palabras.

## Data Availability

Los datos y materiales utilizados para el metaanálisis (incluyendo las coordenadas estandarizadas y parámetros de búsqueda) están disponibles del autor correspondiente previa solicitud razonable.

## Contribuciones de los Autores

EKB: Conceptualización del estudio, diseño metodológico, análisis de datos, redacción del manuscrito, supervisión general. SAGV: Edición y redacción de secciones del manuscrito, análisis de datos y creación de tablas. PTFS: Supervisión académica, revisión crítica del manuscrito, asesoría estadística y metodológica, revisión final para publicación. Todos los autores contribuyeron a los cambios editoriales del manuscrito. Todos leyeron y aprobaron el manuscrito final. Todos los autores participaron lo suficiente en el trabajo y aceptaron ser responsables de todos sus aspectos.

## Aprobación Ética y Consentimiento Informado

No aplicable.

## Agradecimientos

Agradecemos a las instituciones afiliadas al University of Texas Health Science Center at San Antonio y al Medical Scientist Training Program of South Texas por su apoyo institucional y acceso a recursos de neuroimagen. Asimismo, agradecemos al equipo del Research Imaging Institute por su apoyo técnico y metodológico.

## Financiación

Este proyecto fue apoyado con fondos de los Institutos Nacionales de Salud – National Institutes of Health (NIH): NIH/NIGMS: South Texas Medical Scientist Training Program (T32 GM113898); NIH/NIMH: R01 Diversity Supplement Grant (R01 MH074457-11S1). El contenido de este estudio es responsabilidad exclusiva de sus autores y no representa necesariamente las opiniones oficiales de los NIH.

## Material Suplementario

El material suplementario asociado con este artículo se puede encontrar, en la versión en línea, en https://doi.org/10.31083/RN46063.
