# Supplementary material for: Study of Successful Emotional Memory Encoding with Functional Magnetic Resonance Imaging: A Meta-Analysis Comparing the Affective Salience of Words Versus Images
Source: Rev Neurol. 2025 Aug 26;80(7):46063. [Article in Spanish] doi: 10.31083/RN46063 (PMC12415888; doi:10.31083/RN46063)
Supplement: Supplementary file 1 [file 1576-6578-80-7-46063-s1.zip › PRISMA_2020_checklist- Kotkowski.docx]

| **Section and Topic** | **Item #** | **Checklist item** | **Location where item is reported** |
| --- | --- | --- | --- |
| **TITLE** | | |  |
| Title | 1 | Identify the report as a systematic review. | Manuscript Title |
| **ABSTRACT** | | |  |
| Abstract | 2 | See the PRISMA 2020 for Abstracts checklist. | 1 |
| **INTRODUCTION** | | |  |
| Rationale | 3 | Describe the rationale for the review in the context of existing knowledge. | 2 |
| Objectives | 4 | Provide an explicit statement of the objective(s) or question(s) the review addresses. | 2 |
| **METHODS** | | |  |
| Eligibility criteria | 5 | Specify the inclusion and exclusion criteria for the review and how studies were grouped for the syntheses. | 4 |
| Information sources | 6 | Specify all databases, registers, websites, organisations, reference lists and other sources searched or consulted to identify studies. Specify the date when each source was last searched or consulted. | 4 |
| Search strategy | 7 | Present the full search strategies for all databases, registers and websites, including any filters and limits used. | 4 |
| Selection process | 8 | Specify the methods used to decide whether a study met the inclusion criteria of the review, including how many reviewers screened each record and each report retrieved, whether they worked independently, and if applicable, details of automation tools used in the process. | 4 |
| Data collection process | 9 | Specify the methods used to collect data from reports, including how many reviewers collected data from each report, whether they worked independently, any processes for obtaining or confirming data from study investigators, and if applicable, details of automation tools used in the process. | 3,4 |
| Data items | 10a | List and define all outcomes for which data were sought. Specify whether all results that were compatible with each outcome domain in each study were sought (e.g. for all measures, time points, analyses), and if not, the methods used to decide which results to collect. | 4,5 |
|  | 10b | List and define all other variables for which data were sought (e.g. participant and intervention characteristics, funding sources). Describe any assumptions made about any missing or unclear information. | 4 |
| Study risk of bias assessment | 11 | Specify the methods used to assess risk of bias in the included studies, including details of the tool(s) used, how many reviewers assessed each study and whether they worked independently, and if applicable, details of automation tools used in the process. | This type of evaluation was not performed as it was a replication of a previous study (See response section at the end of this document) |
| Effect measures | 12 | Specify for each outcome the effect measure(s) (e.g. risk ratio, mean difference) used in the synthesis or presentation of results. | 4 |
| Synthesis methods | 13a | Describe the processes used to decide which studies were eligible for each synthesis (e.g. tabulating the study intervention characteristics and comparing against the planned groups for each synthesis (item #5)). | 4 |
|  | 13b | Describe any methods required to prepare the data for presentation or synthesis, such as handling of missing summary statistics, or data conversions. | 4 |
|  | 13c | Describe any methods used to tabulate or visually display results of individual studies and syntheses. | 5 |
|  | 13d | Describe any methods used to synthesize results and provide a rationale for the choice(s). If meta-analysis was performed, describe the model(s), method(s) to identify the presence and extent of statistical heterogeneity, and software package(s) used. | 4 |
|  | 13e | Describe any methods used to explore possible causes of heterogeneity among study results (e.g. subgroup analysis, meta-regression). | 4 |
|  | 13f | Describe any Nivity analyses conducted to assess robustness of the synthesized results. | Not applicable (See response section at the end of this document) |
| Reporting bias assessment | 14 | Describe any methods used to assess risk of bias due to missing results in a synthesis (arising from reporting biases). | Discussion: did not include more recent studies, as this was more of a replication analysis (See response section at the end of this document) |
| Certainty assessment | 15 | Describe any methods used to assess certainty (or confidence) in the body of evidence for an outcome. | 4 |
| **RESULTS** | | |  |
| Study selection | 16a | Describe the results of the search and selection process, from the number of records identified in the search to the number of studies included in the review, ideally using a flow diagram. | 3,4 |
|  | 16b | Cite studies that might appear to meet the inclusion criteria, but which were excluded, and explain why they were excluded. | Not applicable (See response section at the end of this document) |
| Study characteristics | 17 | Cite each included study and present its characteristics. | 3 |
| Risk of bias in studies | 18 | Present assessments of risk of bias for each included study. | This type of evaluation was not performed as it was a replication of a previous study (See response section at the end of this document) |
| Results of individual studies | 19 | For all outcomes, present, for each study: (a) summary statistics for each group (where appropriate) and (b) an effect estimate and its precision (e.g. confidence/credible interval), ideally using structured tables or plots. | 5,6,7 |
| Results of syntheses | 20a | For each synthesis, briefly summarise the characteristics and risk of bias among contributing studies. | 6,7 |
|  | 20b | Present results of all statistical syntheses conducted. If meta-analysis was done, present for each the summary estimate and its precision (e.g. confidence/credible interval) and measures of statistical heterogeneity. If comparing groups, describe the direction of the effect. | 4,5 |
|  | 20c | Present results of all investigations of possible causes of heterogeneity among study results. | 6,7 |
|  | 20d | Present results of all sensitivity analyses conducted to assess the robustness of the synthesized results. | Not applicable (See response section at the end of this document) |
| Reporting biases | 21 | Present assessments of risk of bias due to missing results (arising from reporting biases) for each synthesis assessed. | Not applicable (See response section at the end of this document) |
| Certainty of evidence | 22 | Present assessments of certainty (or confidence) in the body of evidence for each outcome assessed. | Not applicable (See response section at the end of this document) |
| **DISCUSSION** | | |  |
| Discussion | 23a | Provide a general interpretation of the results in the context of other evidence. | 6 |
|  | 23b | Discuss any limitations of the evidence included in the review. | 7 |
|  | 23c | Discuss any limitations of the review processes used. | 7 |
|  | 23d | Discuss implications of the results for practice, policy, and future research. | 6,7 |
| **OTHER INFORMATION** | | |  |
| Registration and protocol | 24a | Provide registration information for the review, including register name and registration number, or state that the review was not registered. | Not applicable (See response section at the end of this document) |
|  | 24b | Indicate where the review protocol can be accessed, or state that a protocol was not prepared. | Not applicable (See response section at the end of this document) |
|  | 24c | Describe and explain any amendments to information provided at registration or in the protocol. | Not applicable (See response section at the end of this document) |
| Support | 25 | Describe sources of financial or non-financial support for the review, and the role of the funders or sponsors in the review. | Acknowledgement of NIH Funding |
| Competing interests | 26 | Declare any competing interests of review authors. | No conflicts of interest declared |
| Availability of data, code and other materials | 27 | Report which of the following are publicly available and where they can be found: template data collection forms; data extracted from included studies; data used for all analyses; analytic code; any other materials used in the review. | Public materials available upon reasonable request from the authors |

*From:*  Page MJ, McKenzie JE, Bossuyt PM, Boutron I, Hoffmann TC, Mulrow CD, et al. The PRISMA 2020 statement: an updated guideline for reporting systematic reviews. BMJ 2021;372:n71. doi: 10.1136/bmj.n71. This work is licensed under CC BY 4.0. To view a copy of this license, visit <https://creativecommons.org/licenses/by/4.0/>

**Item #11 – Study risk of bias assessment**

**Reasoning:** This study is a proof-of-concept reanalysis of a previously validated dataset from Murty et al. (2011). As such, we did not re-evaluate the risk of bias for each original study. Our intent was not to reweigh the evidence or make new clinical recommendations but rather to demonstrate the utility of ALE-based meta-analytic tools in extracting lateralization patterns from existing activation maps.

**Item #13f – Sensitivity analyses**

**Reasoning:** As our analysis was designed to explore conceptual and methodological feasibility using a constrained and pre-selected dataset (i.e., studies already included in Murty et al., 2011), we did not perform formal sensitivity analyses. Future iterations of this project may involve a broader inclusion of studies and apply sensitivity methods to test the robustness of the observed lateralization patterns.

**Item #14 – Reporting bias assessment**

**Reasoning:** Because the goal of the present study was to replicate and reinterpret the Murty et al. dataset with a novel analytic focus – rather than generate comprehensive conclusions across all available literature – we did not evaluate publication or reporting bias. We acknowledge this limitation and clarify it in the discussion.

**Item #16b – Excluded studies**

**Reasoning:** We acknowledge that a formal listing of excluded studies is often critical in systematic reviews. However, this work used a fixed dataset ­– comprised entirely of studies from Murty et al. (2011) – as a methodological starting point. We did not apply inclusion/exclusion decisions beyond those made in that original meta-analysis.

**Item #18 – Risk of bias in individual studies**

**Reasoning:** As previously stated under Item #11, our analysis was not structured as an independent systematic review but as a conceptual extension of prior work. Each included study had already passed peer review and quality filters for inclusion in Murty et al.'s study. While future broader meta-analyses may warrant individual bias scoring, it was outside the scope of the current study design.

**Item #20d – Sensitivity analyses of synthesized results**

**Reasoning:** Given the exploratory nature of this reanalysis, we focused on demonstrating differential activation patterns between image and word stimuli. We agree that sensitivity testing could strengthen future conclusions, particularly when including a wider array of studies or conducting subgroup analyses based on sample characteristics or scanner strength. This is a priority for future work.

**Item #21 – Reporting biases in syntheses**

**Reasoning:** Due to the limited and fixed dataset used (derived directly from a prior publication), a formal assessment of publication or outcome reporting bias across syntheses was not feasible. This is acknowledged in our discussion as a methodological limitation of the current proof-of-concept study.

**Item #22 – Certainty of evidence**

**Reasoning:** As this work was not intended to inform policy or clinical guidelines, we did not apply formal frameworks such as GRADE to evaluate certainty. Rather, the goal was to assess the potential of meta-analytic ALE techniques to uncover lateralization signatures in emotional memory. This is acknowledged in the manuscript’s conclusions as a limitation of scope.

**Item #24a–c – Registration and protocol**

**Reasoning:** As this project did not aim to comprehensively or systematically evaluate all available evidence on emotional memory encoding – but instead served as a methodological exploration using pre-existing meta-analytic inputs – we did not prospectively register the review or create a standalone protocol. We agree this would be essential in a broader, future meta-analysis.
